# The prevalence of sexual compulsivity and its correlates among adults living with HIV/AIDS attending antiretroviral therapy clinic in Gambella town, Southwest Ethiopia, 2020

**DOI:** 10.1186/s12888-022-03688-7

**Published:** 2022-01-07

**Authors:** Seid Shumye, Chalachew Kassaw, Getnet Melaku

**Affiliations:** 1 Department of Psychiatry, College of Health and Medical Science, Dilla University, Dilla, Ethiopia; 2 Department of Midwifery, College of Health and Medical Science, Dilla University, Dilla, Ethiopia

**Keywords:** Sexual compulsivity, HIV/AIDS, Gambella, Ethiopia

## Abstract

**Introduction:**

Sexual compulsivity is a concealed psychiatric disease marked by intrusive thoughts followed by ritualized sexual acts. In Ethiopia, the prevalence of HIV/AIDS has recently increased. Furthermore, sexual compulsivity among adults living with HIV/AIDS receives less attention, particularly in Ethiopia. Therefore, this study aimed to assess the prevalence of sexual compulsivity and its correlates among adults living with HIV/AIDS attending ART clinic in Gambella town, Southwest Ethiopia, 2020.

**Method:**

A hospital-based study employing cross-sectional design and simple random sampling technique was used to select the study participants. Data were collected by using interview technique. A 10 item Sexual Compulsivity Scale (SCS) questionnaire was used to assess sexual compulsivity. The translated version of the questionnaire was used for data collection. Bivariate and multivariable logistic regression was conducted to determine factors associated with the outcome variable at *p*-value < 0.05 with a 95% confidence interval.

**Result:**

Out of 300 respondents, 27% (24.3, 29.2) of them were scored above the mean score of the Sexual Compulsivity Scale. Age less than 31 years old, widowed, involving in risky sexual behaviors, current substance use, not received any skill training about safer sex behaviors, and not attending support group discussion on HIV prevention were significantly associated with sexual compulsivity.

**Conclusion:**

Almost one fourth of the respondents have high score for Sexual Compulsivity Scale score. Therefore, there is a need of routine sexual behavior screening program and collaboration with mental health workers for addressing the problem. Furthermore, the emphasis should be given on the identified high-risk categories.

## Introduction

Sexual intercourse is a sexual sensation and intimate activity that involves the insertion and thrusting of the penis into the vaginal canal for sexual pleasure, reproduction, or both. It can be done alone, between two people, or in groups [1]. Humans are compelled to have sex with their sexual partners for biological and psychological reasons [2]. Sexual intercourse boosts the immune system, relieves depression, promotes bladder control and cardiovascular fitness, lowers blood pressure, and improves self-esteem [3, 4].

Sexual compulsivity, also known as sexual dependency, hyper-sexuality, excessive sexuality, or problematic sexual behavior, is defined by persistent and intense problems with sexual desires, impulses, and painful behavior in humans, as well as a psychosocial deficit [5].

Human Immunodeficiency Virus, Acquired Immunodeficiency Syndrome (HIV/AIDS) is one of the most frequent chronic medical disorders spread from person to person through unprotected sexual contact, particularly in Sub-Saharan African nations including Ethiopia [6]. According to the 2016 Ethiopian demography and health survey (EDHS), the Gambella region (4.8%) and Addis Ababa (3.4%) have the highest HIV prevalence rates, while Somali (0.1%) and Southern Nation, Nationalities and People (0.4%) regional states have the lowest rates [7]. Patients with HIV/AIDS have a higher level of sexual desire and conduct than those who are HIV-negative [8]. Patients with HIV/AIDS in low and middle-income countries have a low awareness of safe sexual behaviors and activities, which are critical for preventing transmission from infected to non-infected individuals [9]. The social and self-stigma associated with marriage and sexual partners has been observed among HIV/AIDS patients, contributing to risky sexual behavior. For such risky individuals who transfer the disease to another sexual partner with seronegative status, training the varied safest sexual behaviors such as using condoms, dolls, traps, and masturbations are the safest indicated [10].. Individuals with a higher sexual compulsivity score had more sex partners, engaged in more sexual risk behaviors with casual or one-time sex partners, and were roughly four times more likely to have recently been diagnosed with multiple STIs [11, 12]. Compulsive sexual activity was also linked to the use of alcohol and other drugs [13].

Despite the above facts and the rapidly rising incidence of seropositive people in Sub-Saharan African countries like Ethiopia, where unprotected sexual intercourse is the mainstay of transmission, discussing sexual conduct is fraught with sociological, cultural, and religious taboos, all of which contribute to the spread of a virus [14]. The country’s health ministry and a specialized non-governmental group concentrate on preventing virus transmission, with a focus on condom use. However, there is a lack of research on how to measure and handle patients’ sexual compulsivity, demanding a joint effort from all stakeholders participating in the prevention program. As far as we know, there hasn’t been a published article on this burning issue. Therefore, this study aimed to assess sexual compulsivity and its correlates among adults living with HIV/AIDS attending ART clinic in Gambella town, Southwest Ethiopia, 2020.

## Methods and materials

### Study setting, design and period

A hospital-based cross-sectional study design was conducted at Gambella town ART clinics from March to April, 2020. The town is 778 km away from the capital city, Addis Ababa. It is divided into five Kebeles, each with 12,928 houses and 59,468 population. In the town, there are three government health institutions and 12 private clinics. Only two of the facilities offer ART, and on average a total of 2, 567 customers were actively following their therapy.

### Eligibility Criteria

The inclusion criteria of the current study were adults (age18 years or above) living with HIV/AIDS and attending antiretroviral therapy in the selected health institutions of Gambella town. Respondents with acute or severe physical and mental illness during the data collection period were excluded from the study.

### Sample Size and Sampling Technique

A total of 300 participants from the town’s two ART clinics were involved in the current study. On a monthly basis, a total of 1352 consumers (768 in the first health center and 584 in the second) attended ART clinics. To get the required number of samples, proportional allocation (768*300/1352 = 170, 584*300/1352 = 130) was utilized. Finally, data was collected using a random sampling technique until the needed sample size was attained.

### Data Collection and Instruments

The data was collected by five health professionals working in the ART clinic using a structured interview technique. The first part of the questionnaire was about the socio-demographic of the respondents. The second part was about the respondent’s sexual compulsivity. The validated Sexual Compulsivity Scale (SCS), which has strong acceptability and psychometric properties, was employed in this study. The Sexual Compulsivity Scale (SCS) was developed to assess two characteristics of sexuality: hypersexuality and sexual preoccupation, with items indicating “extreme preoccupation” with sexual behaviors and encounters. Individuals who score at or above the mean score were considered as sexually compulsive. Each SCS item is scored on a four-point scale ranging from 1 to 4 (never applies to me) with a minimum of 10 and a maximum of 40. The sum of all components is used to calculate a total score. With a Cronbach’s alpha of 0.87, it has strong internal validity and reliability [15]. As a result, Sexual Compulsivity Scale were found to be reliable, valid, and helpful in predicting HIV-related risk behaviors. The third part of the questionnaire was factors associated with the out-come variables such as; The PHQ-9 is a nine-item depression screening instrument that detects the presence and frequency of the DSM-V’s core depression symptoms during the past 2 weeks. Scores vary from 0 to 27, with a score of 10 or higher usually indicating the existence of a depressive disorder that requires treatment. The PHQ-9 screening instrument comprises nine items and was validated in Ethiopia with an 86% sensitivity and a 67% specificity [16]. The social support level of study participants was measured using the Oslo Social Support Scale. For epidemiological and population-based surveys, the OSSS-3 has been recommended. The tool comprises three questions that are used to determine the number of people who are close enough to be counted during major personal issues, the level of interest and worry that people display, and the availability of practical support from neighbors if needed, respectively. The first item includes four responses ranging from 1 (none) to 4 (more than five), but the second and third questions each have five responses. The OSSS-3 overall score ranges from 3 to 14. The overall score with a greater value indicates higher levels of social support, and vice versa. The level of social support is also classified into three levels based on the OSSS-3 total score (poor = “3–8,” moderate = “9–11,” and strong = “12–14”) [17]. With acceptable sensitivity and specificity, the tool has been employed in a variety of research. The GAD-7 scale is a seven-item questionnaire designed to assess the severity of anxiety symptoms experienced by participants in the past 2 weeks. The GAD-7 is based on the DSM-5’s concept of anxiety symptoms. Each item is scored on a Likert scale ranging from 0 (not at all) to 4 (very) (nearly every day). All of the components’ scores are combined together to provide a final score that ranges from 0 to 21. Respondents scored > 5/21 was considered as having General anxiety disorder. Test-retest reliability, diagnostic validity, convergent validity, factorial validity, and internal consistency are all acceptable psychometric properties of the GAD-7 in various populations [18]. Adherent to ART medication was assessed using ratio scale of adherence measurement. Those respondents scored above 95% of the division taken number of pills / pills to be taken * 100 [19].

The current substance use history was assed using ASSIST version 2.0 and those who has recent 1 month history of any substance use was considered as current substance user [20].

The 10-item perceived HIV stigma measure, which consisted of four-point Likert scale items (1 = strongly disagree, 2 = disagree, 3 = agree, 4 = highly agree) regarding their HIV status, was used to collect the outcome variable, HIV-related perceived stigma felt by HIV patients. The Cronbach alphas of the 10-item perceived scale ranged from 0.86 to 0.94, and they were tested in a variety of settings, languages, and populations [21, 22]. Those with score of above the mean considered as having perceived stigma [23].

CD4 count, stage of the illness and recent history of STI diagnosis was recorder from the patients’ medical chart. Risky sexual practice, operationalized as engaged at least in one of the following practices such as condom-unprotected sex with any sexual partner, having two or more sexual partners and casual sex in the last 3 months prior to the date of data collection [24].

HIV disclosure status, Attending support group discussion on HIV prevention and receiving any skill training on safer sex behaviors were assed using safest sexual practice guideline [25].

### Data Quality Control

To assure the data quality and consistency, the English version of the questionnaire was translated to Amharic and the local language of the study area and then back-translated to English by a language expert. The pretest study was done on 5% population of near town. Both data collectors and investigators checked data for its completeness daily.

### Data Management and Analysis

The collected data was entered into Epi-data 3.4 and then exported to SPSS (Statistical Package for Social Science 25 version). Descriptive statics such as frequency, percentage, mean and standard deviation were used to describe the socio-demographic and clinical results of the respondents. Variables with *P*-value of less than 0.25 were entered together to the multivariate logistic regression analysis. A binary logistic regression analysis at *p* ≤ 0.05, 95% CI was used to interpret the association between the independent and dependent variables.

## Result

### Socio-demographic results of respondents

The mean (SD) age of respondents was 31 (±4) years old. More than half of them were living in urban areas, and one thirds of the respondents had no formal education (Table 1).

**Table 1:** Socio-demographic characteristics of respondents (*N* = 300)

Variables	Category	Frequency	Percentage (%)
Age	Below 31	177	59%
	Above 31	123	41%
Sex	Male	112	37.3%
	Female	190	63.3%
Educational status	Non-formal	105	35%
	Formal	195	65%
Marriage	Divorced	14	4.8%
	Single	16	5.2%
	Widowed	86	28.8%
	Married	181	60.4%
Residence	Urban	254	84.5%
	Rural	46	15.5%
Religion	Protestant	79	26.2%
	Muslim	29	9.8%
	Orthodox	192	64%
Occupations	Employed	204	68%
	Un employed	96	32%
Income	< 500	171	57%
	> 500	129	43%
Family size	< 3	198	66%
	> 3	102	34%

### Psychosocial and clinical related factors of respondents

Out all of the respondents, 87(29%) and 129(43%) of them had depression and anxiety symptoms, respectively. Among all respondents, 200(66.8%) of them had scored < 500 CD4 count. Those respondents with the mean score of perceived stigma scale 31/50 or 35% had perceived stigma (Table 2.)

**Table 2:** Psychosocial and clinical related factors of respondents (N = 300)

Variables	Categories	Frequency	Percentage
Depression	Yes	87	29%
	No	213	71%
General Anxiety Disorder	Yes	129	43%
	No	171	57%
Current Substance use	Yes	81	27%
	No	219	73%
Adherent to ART	Yes	234	78%
	No	66	22%
Current stage of illness	Stage I & II	97	32.3%
	Stage III & IV	203	67.7%
CD4 cell – count	< 500 cells/mm3	200	66.8%
	> 500 cells/mm3	100	33.2%
Perceived stigma	Yes	105	35%
	No	195	65%
HIV disclosure status	Yes	126	42%
	No	174	58%
Attending support group discussion on HIV prevention	Yes	219	73%
	No	81	27%
Recent history of STI diagnosis	Yes	66	22%
	No	234	68%
Risky sexual behavior	Yes	111	37%
	No	189	63%
Receiving any skill training on safer sex behaviors	Yes	75	25%
	No	225	75%
Social support	Poor	126	42%
	Moderate	75	25%
	Strong	99	33%
Duration of the illness	< 5 year	162	54%
	> 5 year	138	46

### Magnitude of compulsive sexual behavior of the respondents

The mean score for sexual compulsivity scale was 17/40. Out of all, 27% (24.3, 29.2) of them scored above the mean and labeled as high sexual compulsivity score.

### Predictor variables of compulsive sexual behavior

During multivariable logistic regression analysis at 95% CI (*p* < 0.05), risky sexual behavior, widowed, < 31 years old, not receiving any skill training on safer sexual behaviors, current substance use and not attending support group discussion on HIV prevention were associated with the outcome variable (Table 3).

**Table 3:** Variables associated with sexual compulsivity, May 2020 (N = 300)

Variables	Category of variables		SCS		Crude odd ratio (COR)	*P*-value	Adjusted odds ratio (AOR)	*P*-value
			H	L				
RSB	Yes	111	71	40	2.47 (1.52, 4.01)	0.01*	2.58 (1.59, 4.19)	0.02*
	No	189	79	110	1		1	
Age	< 31	177	122	55	3.66 (2.30, 5.82)	0.00*	3.35 (2.11, 5.31)	0.01**
	> 31	123	54	89	1		1	
Sex	Male	110	71	39	2.02 (1.25, 3.28)	0.06*	1.13 (0.86, 2.22)	0.09
	Female	190	90	100	1		1	
ES	Non-formal	105	63	42	1.86 (1.15, 3.01)	0.09*	0.83 (0.51, 1.34)	0.14
	Formal	195	87	108	1			
Residence	Rural	46	24	22	1.36 (0.73–2.55)	0.45		
	Urban	254	113	141	1			
Occupations	Un employed	96	41	55	0.89 (0.55, 1.45)	0.41		
	Employed	204	93	111	1			
Income	> 500ETB	129	60	69	1.01 (0.64, 1.60)	0.32		
	< 500ETB	171	79	92	1			
Family size	> 3	102	47	55	0.93 (0.57, 1.50)	0.26		
	< 3	198	95	103	1			
Religion	Protestant	79	44	35	1.18 (0.70, 2.00)	0.32		
	Muslim	29	13	16	0.76 (0.35, 1.67)	0.89		
	Orthodox	192	99	93	1			
Marital status	Widowed	87	51	36	1.89 (1.13, 3.17)	0.04*	1.83 (1.09, 3.08)	0.05*
	Married	182	78	104	1			
RSTSSB	Yes	213	167	46	10.1 (5.67, 18)	0.00*	9.00 (5.10,15.88)	0.001***
	No	87	23	64			1	
CSU	Yes	81	60	21	2.27 (1.29, 3.99)	0.01*	2.36 (1.34, 4.14)	0.03*
	No	219	122	97	1		1	
ASGDHIVP	Yes	113	43	70	1		1	
	No	187	108	79	2.23 (1.38,3.59)*	0.02*	2.13 (1.32, 3.43)	0.04*
Depression	Yes	87	40	47	0.81 (0.49,1.34)	0.19		
	No	213	109	104	1		1	
GAD	Yes	129	97	32	1.95 (1.18,3.23)	0.09*	1.77 (0.57, 2.20)	0.11
	No	171	104	67	1		1	
Adherent to ART	Yes	234	109	125	1		1	
	No	66	44	22	2.29 (1.29,4.07)	0.07*	1.82 (0.98, 2.12)	0.09
Current stage of illness	Stage I & II	97	42	55	0.79 (0.48,1.28)	0.35		
	Stage III & IV	203	100	103	1			
CD4 cell – count	< 500 cells/mm3	200	94	106	1			
	> 500 cells/mm3	100	45	55	0.92 (0.57,1.49)	0.27		
Perceived stigma	Yes	105	37	68	0.31 (0.19,0.51)	0.16*	0.67 (0.57, 1.44)	0.32
	No	195	124	71	1		1	
HDS	Yes	126	47	79	0.56 (0.35,0.89)	0.14*	0.79 (0.89, 1.81)	0.17
	No	174	90	84	1		1	
RHSTID	Yes	66	31	35	0.96 (0.56,1.67)	0.39		
	No	234	112	122	1			
Social support	Poor	126	67	59	1		1	
	Moderate	75	36	39	0.81 (0.46,1.44)	0.10		
	Strong	99	47	52	0.80 (0.47,1.35)	0.19		
DI	< 5 year	162	84	78	1			
	> 5 year	138	72	66	1.01 (0.64,1.60)	0.23		

*SCS* Sexual compulsivity score, *ASGDHIVP* Attending support group discussion on HIV prevention, *GAD* General Anxiety Disorder, *CSU* Current Substance use, *HDS-HIV* disclosure status, *RHSTID* Recent history of STI diagnosis, *RSTSSB* Receiving any skill training on safer sex behavior, *H* High, *L* Low, *DI* Duration of the illness, *RSB* Risky sexual behavior, *COR* Unadjusted odds ratio /Crude odds ratio, *AOR* Adjusted odds ratio, *ES* Educational status, *, *p-*value < 0.05, **, *P-*value < 0.01, ***, *P-*value < 0.001, model fitness-79%, 1-references

## Discussion

Sexual compulsivity has been linked to changes in executive functions, impulsivity, and difficulties in emotional control. HIV/AIDS is primarily transmitted through sexual contact. Screening for sexual compulsivity is required among infected clients in order to comply with the virus’s national and international prevention protocols. Examining sexual compulsivity and its correlates in these populations is therefore critical in order to apply various psychological and pharmacological treatments.

This result demonstrates a higher sexual compulsivity score among adults living with HIV/AIDS in Gambella Region, Ethiopia compared with reports from South Africa and the United States [12, 26, 27]. The discrepancy could be due to their inclusion criteria: they only included men and used a longitudinal study design. However, consistent results were found from research conducted in China, Germany, and Botswana [28–30].

The chances of developing sexual compulsivity were 3.35 times AOR = 3.35 (2.11, 5.31) higher in the under 31-year-old group than in the older population. These findings matched those of Israel, a public university in the Midwest of the United States, and Denmark [31–33]. This might be due to the natural difference of sexual impulses which young’s have immature and primitive sexual impulses, excessive preoccupation with sexual words, sexual body parts and sexual activity contribute for compulsive and risky sexual practices which then lead to the negative mental and physical consequences [34].

When compared to married participants, widowed respondents were 1.83 times AOR = 1.83(1.09, 3.08) more likely to have a higher score for sexual compulsivity. The findings were supported by a research conducted in New Zealand [35]. After the death of their sexual partner, bereaved people’s sexual lives become disrupted. Masturbation, multiple sexual partners, and causal sexual activity will be used to fulfill sexual desire, which is closely linked to compulsive ideas, impulses, and behaviors.

This study found that people who engaged in risky sexual behavior were 2.58 times AOR = 2.58(1.59, 4.19) more likely to feel sexual compulsivity which was consistent with research conducted in the UK and Milwaukee, USA [11, 31, 36]. Sex is similar to taking a drug that makes feel good, happy, and gives a strong sense of pleasure as a result of dopamine release, a neurotransmitter that activates the brain’s reward center. Furthermore, the brain’s reward region adapted to sexuality’s repeating experience, and it became a compulsive behavior.

When compared to their counterparts, those who did not get any skill training about safer sex behaviors had a 9-fold AOR = 9.00(5.10,15.88) higher chance of developing sexual compulsivity. The findings matched those of studies conducted in the United States and China [13, 36, 37]. Self-awareness, emotional adjustment, and education are all important for managing sexual desires and ritualistic habits. Therefore, life skill training is essential. Individuals who are well-versed in various safe sexual practices, such as the use of toys, traps, and masturbations, are also important in resolving recurrent sexual impulses, thoughts, and behaviors [37].

When compared to non-substance users, respondents with a current history of substance use were 2.36 times AOR = 2.36(1.34, 4.14) more likely to acquire sexual compulsivity. This outcome was similar to those studies conducted in China and the United States [28, 38]. According to neuroimaging research, substance abuse causes aberrant neurotransmitter transmission and anatomical changes that contribute to sexual compulsivity [39]. The last predictor variable linked to sexual compulsivity was not attending HIV prevention support group discussions which was consistent with a study conducted in China [28, 29]. Without fear of being judged or stigmatized, peer support discussion provides an opportunity to share information, expertise, and skills about the control mechanism of sexual urges, feelings, and thoughts.

Many mental health professionals utilize the American Psychiatric Association’s Diagnostic and Statistical Manual of Mental Disorders (DSM-5) as a tool for diagnosing mental health issues. Because compulsive sexual behavior lacks its own diagnostic category in the DSM-5, it may be classified as a subset of another mental health disease, such as impulse control disorder or behavioral addiction. Treatment for compulsive sexual behavior typically involves psychotherapy, medications and self-help groups. A primary goal of treatment is to help manage urges and reduce excessive behaviors while maintaining healthy sexual activities. The treatment of sexual compulsivity in HIV/AIDS patients would lower the number of newly infected people.

### Limitation of the study

This study has a few drawbacks that should be mentioned. First, given this was a cross-sectional study, drawing a causal inference should be approached with caution. Furthermore, because the questionnaire surveys were conducted in conjunction with face-to-face interviews, social desirability may have influenced the results, causing participants to be hesitant to give honest replies.

## Conclusion

This study found a higher score of sexual compulsivity among adults living with HIV/AIDS. The outcome variable was also linked to a number of socio-demographic and psychological variables in this study. Therefore, it is important to have a systematic sexuality assessment screening program in place. Furthermore, there should be a collaboration work between mental health services providers and relevant stakeholders (both government and non-government) organizations to combat the rapid spread of virus and its complications. The focus should also be given to the high-risk categories that have been identified.

## Data Availability

The data used for the study was accessible from corresponding author upon reasonable request.

## Abbreviations

AIDS: Acquired Immune Deficiency Syndrome
ART: Antiretroviral Therapy Treatment
CI: Confidence interval
DSM: Diagnostic and Statistical Manual
EDHS: Ethiopian demographic and health survey
HIV: Human Immunodeficiency Virus
IRB: Institutional Review Board
OSSS: Oslo social support scale
PHQ-9: patient health questioner
SCS: Sexual Compulsivity Scale
SD: Standard deviation
SPSS: Statistical Package for Social Science
STI: Sexually transmitted infection
